# Understanding and Predicting Protein Assemblies With 3D Structures

**DOI:** 10.1002/cfg.310

**Published:** 2003-07

**Authors:** Patrick Aloy, Robert B. Russell

**Affiliations:** EMBL Meyerhofstrasse 1 Heidelberg D69117 Germany

## Abstract

Protein interactions are central to most biological processes, and are currently the subject of great interest. Yet despite the many recently developed methods for interaction discovery, little attention has been paid to one of the best sources of data: complexes of known three-dimensional (3D) structure. Here we discuss how such complexes can be used to study and predict protein interactions and complexes, and to interrogate interaction networks proposed by methods such as two-hybrid screens or affinity purifications.


**Comparative and Functional Genomics**
*Comp Funct Genom* 2003; **4**: 410–415.
Published online in Wiley InterScience (www.interscience.wiley.com). **DOI:** 10.1002/cfg.310

**Conference Review**

### Understanding and predicting protein assemblies with 3D structures

Patrick Aloy and Robert B. Russell*
*EMBL, Meyerhofstrasse 1, D69117 Heidelberg, Germany*

*Correspondence to:
Robert B. Russell, EMBL,
Meyerhofstrasse 1, D69117
Heidelberg, Germany.
E-mail: russell@embl.de

Received: 22 May 2003
Revised: 3 June 2003
Accepted: 3 June 2003

### Abstract

**Protein interactions are central to most biological processes, and are currently the subject of great interest. Yet despite the many recently developed methods for interaction discovery, little attention has been paid to one of the best sources of data: complexes of known three-dimensional (3D) structure. Here we discuss how such complexes can be used to study and predict protein interactions and complexes, and to interrogate interaction networks proposed by methods such as two-hybrid screens or affinity purifications. Copyright © 2003 John Wiley & Sons, Ltd.**

**Keywords: protein interactions; complexes; 3D structure; interaction networks; interaction discovery; prediction**

### Introduction

Proteins are social molecules and thus most cellular processes exploit protein–protein interactions. They are currently the subject of great interest and many innovative techniques to identify interactions or complexes have been developed in recent years, such as the two-hybrid and affinity purification systems [8,9,13,25]. Many thousands of novel interactions have been unveiled and this is changing our perception of cellular function. By one estimate, there are more than 80 000 physical, genetic or functional associations between yeast proteins [26] and these are already being used to assemble the first interaction networks [e.g. 23,15]. We can thus say that the determination of protein interaction networks for whole organisms is a major goal for functional genomics. However, to date, only limited attention has been paid to one of the most accurate sources of protein–protein interactions: complexes of known three-dimensional (3D) structure. This is somewhat surprising, as 3D structures can provide key details for understanding protein function, and these are also exciting times for experimental structural biology.

Here, we review several works recently published by our group. We critically examine interaction discovery techniques from a structural point of view. We also present a new method for predicting protein interactions using 3D structures and finally discuss how combining strengths is leading to new and exciting directions for the understanding of complex biological processes.

### Interaction discovery

Two principal techniques have recently been applied to the large-scale discovery of protein interactions. The yeast two-hybrid system is probably the best-known experimental approach. In the GAL4-based system, a transcription factor for a reporter gene is split in two and fused to proteins of interest. In order for the transcription factor to turn on the reporter gene, the two halves must be brought together by an interaction between the proteins of interest. Two-hybrid analyses are often applied to specific pairs of proteins (reviewed in [17]), and more recently, they have been used in genome-wide screens of yeast [13,25] and *Helicobacter pylori* [20] proteins.

Another approach uses affinity purification. Here, the proteins are tagged (i.e. fused) to one or more proteins that permit easy affinity purification.

Copyright © 2003 John Wiley & Sons, Ltd.

Tagged proteins and their interacting partners are co-purified, and then identified, typically by mass spectrometry. Like the two-hybrid system, this has been applied to whole genomes, identifying many thousands of interacting proteins and complexes, by Ho *et al.* [9] and Gavin *et al.* [8].

### Are we seeing the whole picture?

Comparisons between the interactions discovered by different methods show surprisingly few overlaps [5,26]. This effect is even more pronounced when these sets of interactions are compared to complexes of known 3D structure [5]. One explanation is the paucity of data on both sides: both sets are far from comprehensive. Complex 3D structures lag behind complexes identified by other methods, and comparison shows the overlap between different two-hybrid screens with the same genome to be low, suggesting that techniques miss interactions [12]. One is also not comparing like with like: 3D structures come from all species and cellular locations, in contrast to the other techniques that have, at least to date, predominantly considered yeast proteins, and could be biased for methodological reasons towards soluble, intracellular or nuclear proteins. However, inspection of the data suggests that there could still be other reasons (which we discuss below) for the limited intersection.

Comparison of interactions from the different sources [5] also shows that they have different preferences for the type of interactions they comprise. More specifically, all data sets apart from large-scale affinity purifications favour interactions of a transient nature, where both bound and free components of the complex exist naturally in the cell, rather than tight complexes, where components are not thought to function in isolation. On the other hand, complex purifications [8,9] require protein complexes to withstand purification conditions that might disfavour transient interactions. The high-throughput mass spectrometric protein complex identification (HMS–PCI) method [9] detects roughly equal numbers of transient interactions and permanent complexes. This may be due to overexpression of the interacting components, which is not used during tandem affinity purification (TAP) [8], and thus makes the results more akin to the two-hybrid system [16].

The different nature of the interactions found shows that the methods are highly complementary and that all are needed to cover the diverse protein synergies in the cell. It also raises questions about which data can be used to construct protein interaction networks. Tight complexes function as single entities, and should probably be considered as such in a network, since the individual components are not found in isolation.

### Some illustrative examples

Knowledge of protein structure can help to understand the molecular details that take place in protein interactions. Here, we have identified several instances that illustrate effects often not considered when interpreting the results from interaction discovery.

Although the typical diagram demonstrating a two-hybrid experiment suggests that proteins are physically interacting, structures show that some interactions are *indirect*, mediated by one or more endogenous proteins, instead of contacting each other directly. One genome-wide two-hybrid study found interactions between the yeast cyclins Clb1, Clb2 and Clb3 and Cks1 [25]. The human equivalents of these proteins are in separate 3D complexes with cyclin-dependent kinase 2 (CDK2), suggesting that the kinase acts as an intermediary (Figure 1a). Despite the overexpression of the two test proteins, endogenous levels of the intermediaries are apparently sufficient to lead to a two-hybrid signal. This effect is probably more pronounced when the two test proteins normally reside in the nucleus (where the two-hybrid interaction is thought to occur) and when they are themselves from yeast, although interactions between equivalent proteins in species as remote as human and yeast are known (e.g. [19]).

Techniques for interaction discovery require proteins to be fused to others. The two-hybrid system usually involves attaching different functional domains from a transcription factor to the N- or C-terminus of bait and prey proteins, and affinity purification requires a tag to be attached to one or more proteins in the complex. Fusions that place these additional proteins at important interacting interfaces are expected to disrupt normal complex formation. Moreover, some proteins might be unstable when attached to a foreign body and fail to fold: attaching molecular labels necessary

Copyright © 2003 John Wiley & Sons, Ltd. 

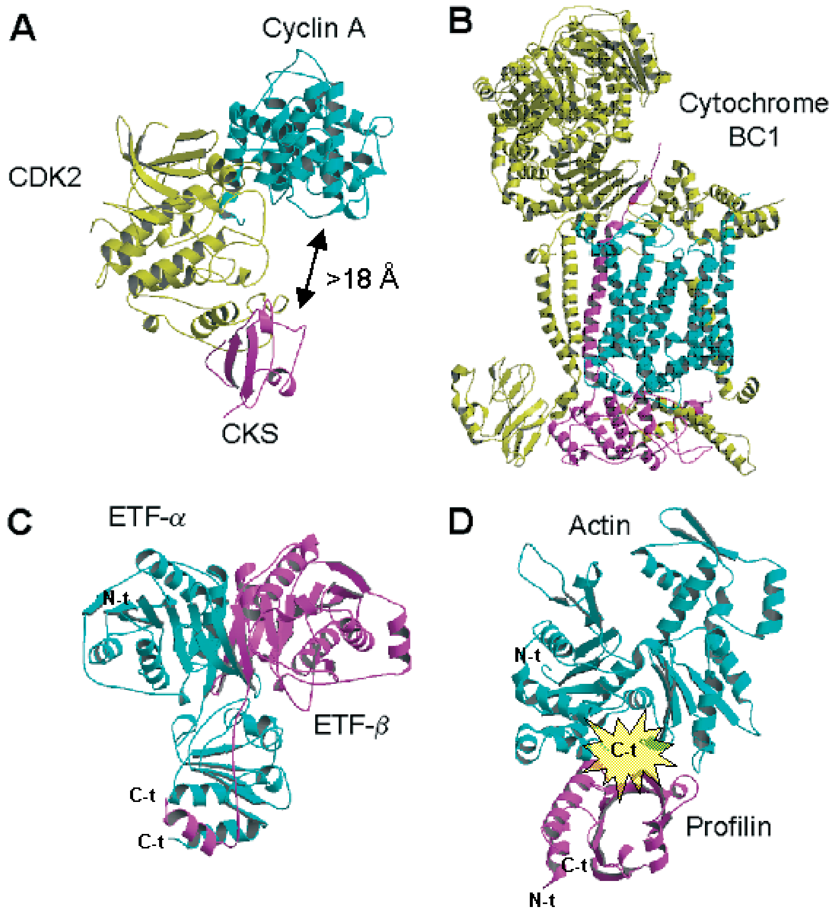

**Figure 1.** Examples of 3D protein interactions and complexes. (A) The human cyclin A/cyclin-dependent kinase 2 (CDK2) complex [14] superimposed (using CDK2) on the human CDK2/cyclin-dependent kinase regulatory subunit (CKS) complex [7]. (B) The yeast cytochrome *bc1* complex [11]. (C) The human electron transfer flavoprotein $\alpha$- and $\beta$-subunits [21]. (D) The bovine $\beta$-actin and profilin complex [22]. The explosion symbol shows how attaching an extra subunit at the C-terminus would disrupt the interaction interface to use a technique might end up disrupting the phenomenon studied.

Two-hybrid interactions were not detected between cytochrome *bc1* components (Figure 1b) or between the electron transfer flavoprotein (ETF) $\alpha$- and $\beta$-subunits (Figure 1c). One possible explanation for the first is that the complex is located in the mitochondrial membrane, meaning that the highly cooperative nature of the interactions cannot be reproduced in the nucleus. No obvious explanation for the second is apparent: this is simply a known interaction between soluble proteins that is not detected during a two-hybrid screen.

There are examples of protein complexes of known 3D structure where the N- or C-terminus of the components lies at the interaction interface, and thus where one might predict the attachment to a foreign protein to be disruptive. The C-terminus of profilin lies at the interface of its interaction with actin (Figure 1D). One would predict a two-hybrid experiment that attaches part of a transcription factor to this terminus to result in a false negative (i.e. a true interaction that would be missed by the screen).

Evidence of interference also comes from the observation that different tagged proteins do not always retrieve the same complexes, and some appear to fail altogether (e.g. cytochrome oxidase and cytochrome *bc1* complexes). Other evidence lies in the nature of proteins with many interaction partners found in the large-scale complex affinity purification screens [8,9]. Two-thirds of the TAP,

Copyright © 2003 John Wiley & Sons, Ltd. 

and half of the HMS–PCI, purifications retrieved at least one protein involved in heat shock, the ribosome or the proteasome, suggesting that the proteins in the complexes have been caught in the process of synthesis, refolding or degradation (Figure 2A, B). The interactions are thus biologically correct, but are occurring because of tinkering with the genome. It is remarkable that many complexes are still able to assemble normally when one or more components are both disrupted owing to the fusion, and bound to other proteins or complexes involved in their formation or destruction.

There are also known sources of errors without structural explanations. One source is those proteins that are able to give a signal as 'bait' with any 'prey' in a two-hybrid experiment. Certain proteins, when fused to the DNA-binding domain, are able to turn on the reporter gene even in the absence of the activation domain (e.g. [10]), possibly because of an acidic 'blob' on the bait that is able to replace the activation domain [18]. One must be wary of those proteins that apparently interact with many others as bait [4].

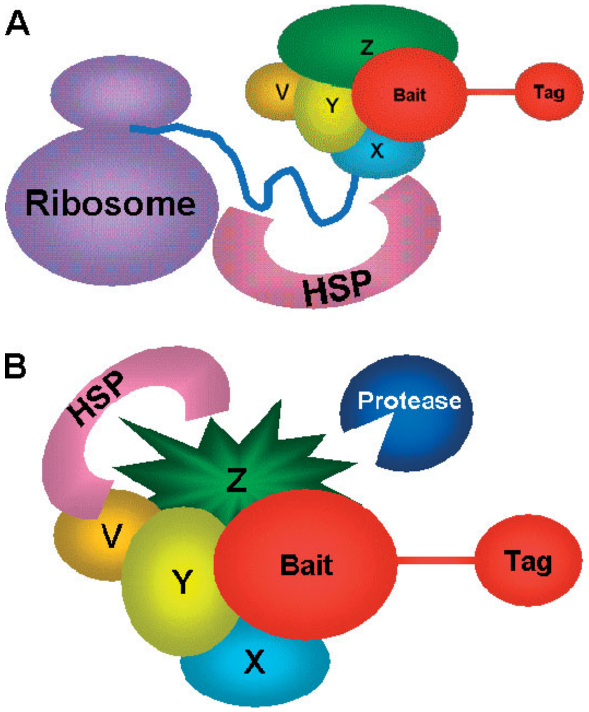

**Figure 2.** Possible explanations for some sticky proteins; two hypothetical scenarios. In (A) one of the complex components is still attached to the ribosome and is also bound to a chaperonin. In (B) a component is misfolded due to interference from the tag, and is bound to a protease and a chaperonin

### Using 3D structures to predict interactions

A feature that we observed in the set of complexes inspected is that interacting pairs of proteins belonging to the same families usually interact in the same way. We first systematically checked whether it is indeed possible to extrapolate interaction details of one protein complex to associated homologues. We then developed a method to model putative interactions on known 3D complexes and to assess the compatibility of a proposed protein–protein interaction with such a complex [3,6]. Briefly, after identifying the residues that make atomic contacts in a known complex structure, we look to homologues of both interacting proteins to see if these interactions are preserved by means of empirical potentials. This permits us to score all possible pairs between two protein families, and say which are likely to interact. We tested the method in the fibroblast growth factor/receptor system, and explored the intersection between all complexes of known 3D structure and interactions between yeast proteins proposed by methods such as two-hybrids.

We applied the method large-scale to 90 completed genomes, including 10 eukaryotes, and predicted with confidence around 23 000 interactions in human (involving 2700 proteins) out of more than 2 million tested. This approach can be used to filter the raw data coming from large-scale proteomics experiments and prioritize experiments, which will save time and money (i.e. from millions of potential interactions down to a few thousands).

### *Complex* structural genomics

Interaction discovery can uncover new interactions and complexes on a genome-wide scale, and has already provided many insights into cellular function. Structural biology, although slower, ultimately provides the critical biological answers: key atomic details of function, and verification of interactions often first identified by other methods. Molecular biology moves towards understanding ever-larger cellular structures, and increasingly involves fusions between these two disciplines.

Copyright © 2003 John Wiley & Sons, Ltd.

However, before entering the *complex* structural genomics era we will have to face new difficulties in addition to those that already hamper existing structural genomics projects (i.e. production of sufficient quantities of protein, difficult crystallization of isolated complex components, etc.)

There is some middle ground, which might be covered by combining complex purification, low-resolution structure determination and computational biology. With smaller quantities of protein, such as those recovered from genome-wide affinity purifications [8,9], it is possible to obtain structures by electron microscopy. Although it is still rare for these structures to reach atomic resolution, they provide valuable shape information. If the components are known (i.e. from the affinity purification screens) and sequence comparisons show homology, or even identity, to known 3D structures, it may be possible to fit these into the low-resolution models. Such efforts will clearly be aided by the current structural genomics efforts that provide structures for individual proteins or their homologues, in effect providing more pieces for the puzzles.

The approach of combining complex discovery/purification, low-resolution EM and computational biology has already been used to produce approximate atomic models for cellular machines such as the *Escherichia coli* ribosome [24] and the yeast exosome [2]. There is now an international consortium to work out a high-throughput strategy for discovering and solving the structures of new protein complexes [1].

### Conclusions

Interaction discovery methods will continue to provide important protein interaction and complex data. A thorough understanding of these techniques and identification of the source of systematic errors would reduce the number of missed or incorrect interactions and eventually lead to cleaner data sets for further use. We can also expect 3D structures for increasingly complicated macromolecular complexes. The time is right for a synthesis of interaction discovery with structural biology. Together they can face the exciting challenge of providing atomic descriptions of complex cellular machineries.

### Acknowledgements

We would like to thank Professor Rita Casadio for the kind invitation to the Bologna Winter School and to write this manuscript. We also thank Elsevier for permission to reproduce text and figures from our *TiBS* paper.

### References

1. Abbott A. 2002. Proteomics: the society of proteins. *Nature* **417**: 894–896.
2. Aloy P, Ciccarelli FD, Leutwein C, *et al.* 2002. A complex prediction: three-dimensional model of the yeast exosome. *EMBO Rep* **3**: 628–635.
3. Aloy P, Russell RB. 2002a. Interrogating protein interaction networks through structural biology. *Proc Natl Acad Sci USA* **99**: 5896–5901.
4. Aloy P, Russell RB. 2002b. Potential artefacts in protein-interaction networks. *FEBS Lett* **530**: 253–254.
5. Aloy P, Russell RB. 2002c. The third dimension for protein interactions and complexes. *Trends Biochem Sci* **27**: 633–638.
6. Aloy P, Russell RB. 2003. InterPreTS: protein interaction prediction through tertiary structure. *Bioinformatics* **19**: 161–162.
7. Bourne Y, Watson MH, Hickey MJ, *et al.* 1996. Crystal structure and mutational analysis of the human CDK2 kinase complex with cell cycle-regulatory protein CksHs1. *Cell* **84**: 863–874.
8. Gavin AC, Bosche M, Krause R, *et al.* 2002. Functional organization of the yeast proteome by systematic analysis of protein complexes. *Nature* **415**: 141–147.
9. Ho Y, Gruhler A, Heilbut A, *et al.* 2002. Systematic identification of protein complexes in *Saccharomyces cerevisiae* by mass spectrometry. *Nature* **415**: 180–183.
10. Housni HE, Vandenbroere I, Perez-Morga D, Christophe D, Pirson I. 1998. A rare case of false positive in a yeast two-hybrid screening: the selection of rearranged bait constructs that produce a functional Gal4 activity. *Anal Biochem* **262**: 94–96.
11. Hunte C, Koepke J, Lange C, Rossmanith T, Michel H. 2000. Structure at 2.3 Å resolution of the cytochrome *bc(1)* complex from the yeast *Saccharomyces cerevisiae* co-crystallized with an antibody Fv fragment. *Structure Fold Des* **8**: 669–684.
12. Ito T, Chiba T, Ozawa R, *et al.* 2001. A comprehensive two-hybrid analysis to explore the yeast protein interactome. *Proc Natl Acad Sci USA* **98**: 4569–4574.
13. Ito T, Tashiro K, Muta S, *et al.* 2000. Toward a protein–protein interaction map of the budding yeast: a comprehensive system to examine two-hybrid interactions in all possible combinations between the yeast proteins. *Proc Natl Acad Sci USA* **97**: 1143–1147.
14. Jeffrey PD, Russo AA, Polyak K, *et al.* 1995. Mechanism of CDK activation revelated by the structure of a cyclinA–CDK2 complex. *Nature* **376**: 313–320.
15. Jeong H, Mason SP, Barabasi AL, Oltvai ZN. 2001. Lethality and centrality in protein networks. *Nature* **411**: 41–42.
16. Kemmeren P, van Berkum NL, Vilo J, *et al.* 2002. Protein interaction verification and functional annotation by integrated analysis of genome-scale data. *Mol Cell* **9**: 1133–1143.

Copyright © 2003 John Wiley & Sons, Ltd. 

17. Legrain P, Wojcik J, Gauthier JM. 2001. Protein–protein interaction maps: a lead towards cellular functions. *Trends Genet* **17**: 346–352.
18. Ma J, Ptashne M. 1987. A new class of yeast transcriptional activators. *Cell* **51**: 113–119.
19. Paris J, Leplatois P, Nurse P. 1994. Study of the higher eukaryotic gene function CDK2 using fission yeast. *J Cell Sci* **107**: 615–623.
20. Rain JC, Selig L, De Reuse H, *et al*. 2001. The protein–protein interaction map of *Helicobacter pylori*. *Nature* **409**: 211–215.
21. Roberts DL, Frerman FE, Kim JJ. 1996. Three-dimensional structure of human electron transfer flavoprotein to 2.1 Å resolution. *Proc Natl Acad Sci USA* **93**: 14 355–14 360.
22. Schutt CE, Myslik JC, Rozycki MD, Goonesekere NC, Lindberg U. 1993. The structure of crystalline profilin-$\beta$-actin. *Nature* **365**: 810–816.
23. Schwikowski B, Uetz P, Fields S. 2000. A network of protein–protein interactions in yeast. *Nat Biotechnol* **18**: 1257–1261.
24. Spahn CM, Beckmann R, Eswar N, *et al*. 2001. Structure of the 80S ribosome from *Saccharomyces cerevisiae*-tRNA–ribosome and subunit–subunit interactions. *Cell* **107**: 373–386.
25. Uetz P, Giot L, Cagney G, *et al*. 2000. A comprehensive analysis of protein–protein interactions in *Saccharomyces cerevisiae*. *Nature* **403**: 623–627.
26. Von Mering C, Krause R, Snel B, *et al*. 2002. Comparative assessment of large-scale data sets of protein–protein interactions. *Nature* **417**: 399–403.

Copyright © 2003 John Wiley & Sons, Ltd. 
